# Impact of a multimodal effort re-education programme on functionality, physical performance, and functional capacity in cancer patients with dyspnoea: a randomised experimental study

**DOI:** 10.1007/s00520-024-08852-1

**Published:** 2024-09-06

**Authors:** Eduado Jose Fernandez-Rodriguez, Celia Sanchez-Gomez, Emilio Fonseca-Sanchez, Juan Jesus Cruz-Hernandez, Maria Isabel Rihuete-Galve

**Affiliations:** 1 Department of Nursing and Physiotherapy, University of Salamanca, Salamanca, Spain; 2 Department of Developmental and Educational Psychology, University of Salamanca, Salamanca, Spain; 3 Department of Medicine, University of Salamanca, Salamanca, Spain; 4 Medical Oncology Service, University Hospital of Salamanca, Salamanca, Spain; 5 Salamanca Biomedical Research Institute (IBSAL), Salamanca, Spain

**Keywords:** Dyspnoea, Cancer patients, Treatments

## Abstract

**Background:**

In recent years, there has been a significant increase in the survival rates of cancer patients. However, this has also led to an increase in side effects, such as dyspnoea, which can negatively impact of patients. We propose a programme for re-educating effort. The main objective is to test the effectiveness of this programme in improving respiratory symptoms and functionality in cancer patients.

**Methodology:**

Experimental, prospective, longitudinal, randomised study with a parallel fixed-assignment scheme (CG-IG). The patients were selected from the Medical Oncology Service of the University Hospital Complex of Salamanca (CAUSA), Spain. Two parallel intervention programmes were designed for the two study groups (Conventional Clinical Practice-Effort Re-education Programme). Primary variables: dyspnoea (MRC), functionality (Barthel); secondary variables: physical performance (SPPB) and functional capacity (ECOG) and the socio-demographic variables (age, sex, anatomopathological diagnosis, and number of treatment lines).

**Results:**

The study sample consisted of 182 patients, with 12 excluded, resulting in a final sample size of *n* = 170. Sex distribution (CG: 52.9% male and 47.1% female; IG: 49.4% male and 50.6% female). The primary oncological diagnosis was lung cancer, and the most frequent tumour stages were III and IV. Statistically significant differences were found between the IG and CG scores (*p* < 0.001, *d* = 0.887, 95% CI) and between the IG and CG scores (*p* = 0.004, *d* = 0.358, 95% CI), indicating that the IG performed better.

**Conclusion:**

The results of this study support the beneficial effects of an exercise re-education programme, carried out by an interdisciplinary team in improving the autonomy of oncology patients with dyspnoea.

**Trial registration:**

The clinical trial was registered in ClinicalTrials.gov (NCT04186754). (03 September 2019).

## Background

In recent years, advancements in oncological treatments, coupled with a dedicated focus on preventive strategies, have resulted in better early diagnosis and understanding of oncological pathology. This has led to an exponential increase in the 5-year survival rate of cancer patients [1].

Alongside the increase in survival rates, the use of multiple treatment lines has led to a rise in negative side effects that can impact patients’ functionality and quality of life. These side effects include tumour asthenia, anxiety, and dyspnoea [2–4]. Dyspnoea, in particular, can be a serious health concern for individuals and may indicate end-stage disease in some advanced cancer patients. Approximately 41% of palliative care patients have the symptom and 46% of them describe it as moderate or severe [5, 6].

Patients with respiratory problems often perceive dyspnoea as a limiting factor beyond their control, leading them to engage in avoidance behaviours that further increase their inactivity. This, in turn, has negative repercussions on their functionality.

Decreasing their level of activity to adapt to their symptomatology leads to a worsening of physical fitness and exertional dyspnoea [7].

To control dyspnoea, non-pharmacological strategies should be implemented alongside conventional clinical practices. Our proposal is to re-educate individuals in their daily life activities from a rehabilitative perspective [8]. The aim of this programme is to enable individuals with significantly reduced cardiopulmonary reserves to manage their daily activities and regain the ability to perform occupational roles within their potential range.

Upon analysis of current studies, it is evident that physical exercise is the most commonly used intervention. This intervention has been shown to have a positive impact on the improvement of dyspnoea [9–13]. However, there is a lack of studies that correlate this symptomatic improvement with an effective impact on patients’ functionality [14–16]. Furthermore, we believe that this intervention is only aimed at symptom improvement, but we consider it important to carry out direct interventions on the autonomy of individuals that allow the symptomatic improvements obtained with the therapeutic process to be generalised in the patient’s daily life. This will inexorably go hand in hand with an improvement in their quality of life.

Additionally, exercise re-education stands out from other interventions due to its holistic and integrative approach. While traditional interventions typically focus solely on symptomatic relief through medication or specific breathing techniques, exercise re-education addresses the root of the problem by improving the patient’s overall physical condition. This not only helps to alleviate the symptoms of dyspnoea but also strengthens the cardiovascular and pulmonary systems, enhances muscular endurance, and promotes greater functional autonomy. By integrating exercises tailored to the individual capabilities of each patient, this strategy allows for a progressive and personalised adaptation, resulting not only in physical improvement but also in increased confidence and psychological well-being. In summary, exercise re-education not only combats symptoms but also prepares the patient for a more active and autonomous life, something that cannot be achieved with more traditional and one-dimensional approaches.

Given the aforementioned considerations, we suggest an interdisciplinary ‘effort re-education programme’ led by a team of occupational therapists, nurses, and oncology doctors. The aim is to evaluate whether this approach can yield better clinical outcomes. The primary objective is to assess the programme’s efficacy in enhancing respiratory symptoms and functionality in oncology patients. Additionally, the study aims to investigate secondary objectives, including improvements in physical performance and functional capacity, as well as analysing the impact of socio-demographic factors on the intervention’s effectiveness.

## Material and methods

### Design

This is an experimental, prospective, longitudinal, randomised, parallel, fixed-assignment study with both experimental and control groups.

### Participants

The Hospitalisation Unit of the Medical Oncology Service at the Complejo Hospitalario Universitario de Salamanca (CAUSA), Spain, treats patients.

### Selection criteria

- **Inclusion criteria:** Inclusion criteria for participation in the study were an anatomopathological diagnosis of oncological disease, admission to the CAUSA Oncology Unit at the time of recruitment, dyspnoea parameters equal to or higher than 2 points on the Medical Research Council (MRC) scale, a score of less than 85 points on the Barthel Index, and voluntary agreement to participate in the study as indicated by signing an informed consent form.
- **Exclusion criteria:** Exclusion criteria for this study included having a clinical diagnosis of cognitive impairment at baseline that interferes with the understanding and performance of the assessment (scores below 23 points on the Mini Mental State Examination, MMSE), haemoglobin levels below 10 g/dl, and current active smoking status at the time of recruitment.
- **Withdrawal criteria:** The patient’s treatment may be discontinued if their condition progresses to a terminal stage or if they pass away.

### Sample size

The sample size was estimated based on the potential for modification of one of the main variables of the study, namely the Barthel Index score. A pilot study was conducted for 3 months (February–May 2019), in which 14 individuals were included to identify the effect of an activity of daily living re-education programme on improving the functionality of cancer patients with dyspnoea.

The study reports a modification of 6.5 points in the score of the main variable under investigation, the Barthel Index. To detect a difference equal to or greater than 6.5 units, 83 subjects are required in each of the two groups, assuming a risk alpha of 0.05 and a risk beta of less than 0.2 in a bilateral contrast. The common standard deviation is assumed to be 13.3, and a loss to follow-up rate of 20% has been estimated.

### Recruitment and randomisation

After passing the selection criteria and completing the initial interview with the research team, the study population will be randomly assigned to either the intervention group (IG) or the control group (CG) in a 1:1 ratio. The allocation sequence will be generated by an independent researcher using Epidat 4.2 software. Participants will be randomised based on the order of their baseline assessment. Those with an even allocation number will be assigned to the intervention group (IG), while those with an odd allocation number will be assigned to the control group (CG). The randomisation sequence will remain hidden until each patient is assigned to the appropriate group.

Following the recruitment of patients based on the selection criteria and their random assignment to respective groups, both groups underwent a baseline assessment (T0) to record the variables under study. During patient admission, the control group received conventional pharmacological clinical practice, while the experimental group participated in the ‘stress re-education’ programme. Each patient in the experimental group received daily 45-min sessions. At the end of the intervention, patients in both groups underwent a final evaluation (T1) before being discharged from the hospital. Recruitment took place between June 2019 and January 2023.

### Blinding

The sequencing, randomisation, recruitment, and allocation of the study sample were carried out by research staff who were not involved in the assessments or interventions of each group. This approach helped to avoid potential biases in the study.

Additionally, the participating subjects were blinded to their assigned group and the intervention they received. To minimise any contamination between groups, external researchers who were previously educated and trained carried out the evaluation process and measurements. This was done to avoid subjective biases in the process. The researchers were unaware of the intervention group to which they were assigned, ensuring a blinded evaluation by third parties. Furthermore, the researchers in charge of the statistical analysis were also blinded to increase the study’s rigour and scientific quality.

The study was conducted with the prior informed consent of the study subjects and in accordance with the Declaration of Helsinki, after authorisation by the Clinical Research Ethics Committee of the Salamanca Health Area. The participants were informed of the objectives of the project and the risks and benefits of the interventions that were carried out. The confidentiality of the included subjects was ensured in accordance with Organic Law 3/2018 of 5 December on Personal Data Protection and guarantee of digital rights, Regulation (EU) 2016/679 of the European Parliament and of the Council of 27 April 2016 on data protection (GDPR), and Law 14/2007 on biomedical research. The approval number is 0000263, and the name of the board is the Bioethics Committee of the University of Salamanca. Board Affiliation: University of Salamanca.

### Description of the variables

The study examined the primary variable of dyspnoea and performance of activities of daily living, as well as the secondary variables of physical performance and functional capacity. Socio-demographic variables such as age, gender, anatomopathological diagnosis of the patients, and the number of treatment lines used were also assessed.

### Tools employed in the evaluation of the variables

Assessment instruments were used to measure functionality, dyspnoea, physical performance, and functional capacity in oncology patients. The Barthel Index (IB) [17] was employed to measure the performance of activities of daily living, with scores ranging from 0 (completely dependent) to 100 (completely independent). The Medical Research Council Dyspnoea Scale (MRC) [18] assessed dyspnoea, with scores ranging from 1 (no dyspnoea) to 5 (severe dyspnoea). The Short Physical Performance Battery (SPPB) [19] evaluated physical performance, with scores from 0 (worst performance) to 12 (best performance). The ECOG Performance Status Scale [20] was used to measure functional capacity in oncology patients, with scores ranging from 0 (fully active) to 5 (dead). The variables were recorded on an individual sheet for each patient and then stored in a database designed specifically for this study.

### Interventions

Two parallel intervention programmes were designed for the two study groups. The first group received Conventional Clinical Practice (CCP), which included pharmacological treatment and a Health Education Programme. The second group received the control group intervention and an Effort Re-education Programme (ERP), following the TIDieR guidelines.

Both programmes were structured and supervised by the research team at the University of Salamanca (Spain) (Table 1).

**Table 1:** Detailed explanation of the interventions used in both study groups

**A. Control Group (CG): Conventional Clinical Practice: Pharmacological Treatment + Health Education Programme**
*Short name: Health Education Programme*
Why: Upon admission to the hospital, the medical team prescribed pharmacological treatment based solely on the patient’s symptoms. The nursing team provided instructions and recommendations for maintaining a healthy lifestyle, promoting self-care and good care practices as part of a health education programme. The programme focused on the benefits of an active lifestyle and general guidelines to follow
What (materials): The necessary drugs for symptomatology control and recommendations from the health education programme were used, through an information dossier
What (procedures): Instructions and recommendations were provided to promote self-care and good care practices as part of a health education programme aimed at maintaining a healthy lifestyle. The participants received a dossier containing the instructions and recommendations of the programme
Who will carry out the interventions: The treatment interventions were performed by an experienced and qualified team member
How: The material was given to each patient individually upon admission
Where: All sessions were held at the Complejo Asistencial Universitario de Salamanca (CAUSA)
When and for how long: The intervention was administered daily during hospitalisation, with participants receiving information on the timing and duration
Adaptation: Adaptations will also be available. Due to the diversity of topics and the individual nature of the intervention, sessions will be adapted to each topic
How well (planned): Supervision of the therapy will take place through weekly meetings between therapists and researchers. The sessions were held twice a week
**B. Intervention Group (IG): Effort Re-education programme for improving performance in activities of daily living**
*Short name: Exercise re-education programme*
Why: The programme is an interdisciplinary non-pharmacological intervention that combines occupational therapy, nursing, and oncological medicine. Its aim is to meet the needs of cancer patients by providing training in knowledge and strategies for the correct execution of daily activities, promoting maximum autonomy and functionality. The intervention is based on the principle of complementing and integrating both disciplines
What (materials): The required materials for all sessions include mobility aids such as walkers, canes, or wheelchairs, support products to preserve the integrity of the patient’s tissues such as anti-decubitus cushions, heel supports, wedges, and other devices for the correct positioning of the patient, and support products for daily activities such as dressing, personal hygiene, and feeding, as well as an incentive spirometer
What (procedures): Stress re-education programme: The intervention involved ‘stress re-education’ and was conducted by an interdisciplinary team of occupational therapists and nurses on individuals. The following interventions were implemented:
1. Progressive mobilisation
An individualised assessment was carried out in which support products were prescribed to facilitate the patient’s mobilisation (walker with adaptation for oxygen therapy, portable oxygen concentrator for activities of daily living)
Additionally, a comprehensive daily record was kept of the patient’s activities, which was then modified to suit their clinical situation. This established a suitable pattern that enabled the patient to improve their autonomy by utilising all their present capacities without exceeding them. This was accomplished using a daily record sheet that the patient completed and the occupational therapist monitored
2. Gradation and Simplification of Activities of Daily Living: Teaching Techniques to Save Energy
Individual training was conducted on rules for simplifying activities. The training consisted of:
- Organising work areas exhaustively, adapting work plans - Adapting work plans, placing objects within the patient’s reach - Prioritising basic self-care activities while seated - Controlling movement with slow, coordinated, and harmonious movements while avoiding impulsive and vigorous movements - It is recommended to alternate heavy activities with light activities and establish appropriate rest periods between them - Additionally, it is suggested to combine periods of balanced activity with periods of rest
3. Breathing exercises and incentive spirometry
Incentive spirometry (IS) is a mechanical treatment technique used to reduce pulmonary complications during postoperative care. The nurse performed this technique, taught the patient, and monitored them in collaboration with the physician
In addition to this intervention, patients in this group also received the same interventions as the control group, which included pharmacological treatment and a Health Education Programme
Who will carry out the interventions: All interventions were performed by qualified occupational therapists, nurses, and medical specialists with experience in treating cancer patients
How: The technique used was individual
Where: The sessions will take place in person and individually at the Hospitalisation Unit of the Medical Oncology Service of the Complejo Asistencial Universitario de Salamanca, Spain
When and for how long: Each participant received one individual session per day during their hospitalisation period. The duration of each session was 1 h
Adaptation: The sessions were tailored to each patient individually in terms of complexity due to the clinical diversity and individual nature of the intervention
How well (planned): The intervention programme was planned and monitored through weekly meetings attended by all research members. Two sessions were held twice a week, and subject attendance was closely monitored

Additionally, as this is a randomised clinical trial, it has been registered. TRIAL REGISTRATION: ClinicalTrials.gov NCT04186754; https://register.clinicaltrials.gov/prs/app/action/SelectProtocol?sid=S0009702&selectaction=Edit&uid=U0004OJ7&ts=2&cx=-hsiipg

### Statistical analysis

The statistical analysis was pre-planned with minor modifications made once the study was carried out. Initially, we conducted an exhaustive review and filtered the data to detect possible errors in data collection. This was done to ensure the correct and accurate application of the exclusion criteria established for the study. The statistical analysis will be conducted using the intention-to-treat approach. The normal distribution of all variables was checked using the Kolmogorov–Smirnov test (*p* < 0.05), and reflected in a histogram. A descriptive analysis of the variables was then carried out. Quantitative variables were expressed as median and interquartile range, while qualitative variables were defined by frequencies and percentages.

Analytical statistics were conducted using the Wilcoxon test to compare means between groups. Effect sizes (ES) of the experimental treatment were calculated using Cohen’s *d* values (small (0.2), medium (0.5), and large (0.8)). Correlations between the different intervening variables and the parameters established in the different questionnaires and scales used were analysed using Spearman’s correlation coefficient. A confidence interval of 95% was used to determine statistical significance, with *p*-values less than 0.05 considered significant. The statistical analysis was performed using IBM SPSS Statistics version 28.0.1.

## Results

**Fig. 1:**
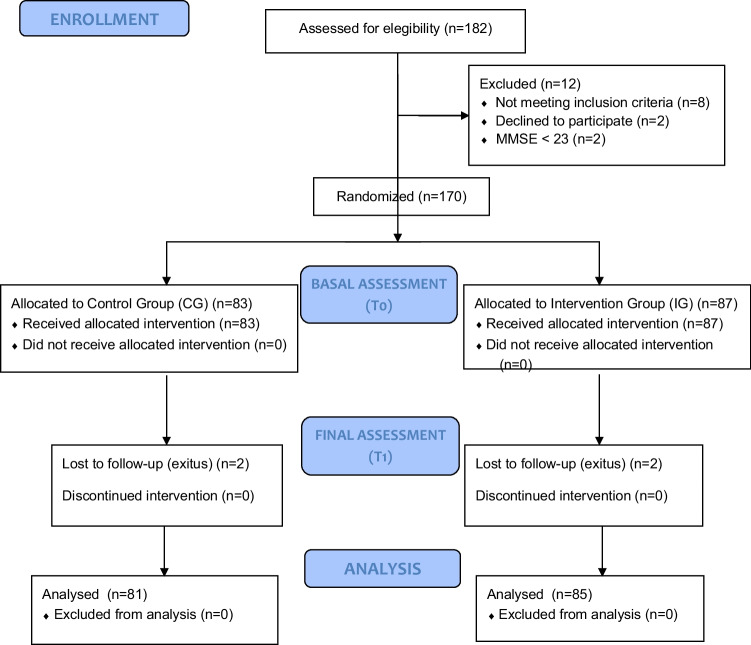
CONSORT flow diagram of participants included on the trial

The study sample consisted of 182 patients, of which 12 were excluded. The remaining 170 patients were randomly assigned to two groups, as shown in Fig. 1. The intervention group consisted of 87 participants, while the control group consisted of 83 participants. However, two participants from each group did not continue with the intervention due to reported health problems. There were no statistically significant differences between the groups at baseline (*p* > 0.05) in any of the demographic and clinical data recorded (refer to Table 2). Additionally, no adverse effects of the intervention protocol were detected.

**Table 2:** Demographic and clinical data baseline assessment (median and range for continuous data and frequencies (%) for categorical data)

Variables		IG (*N* = 85)	CG (*N* = 81)
Age		67 (42)	67 (41)
Sex	Male	52.9%	49.4%
	Female	47.1%	50.6%
Pathological diagnosis	Breast	12.9%	17.3%
	Lung	61.2%	59.3%
	Digestive system	20.0%	18.6%
	Central nervous system	2.4%	1.2%
	Prostate	1.2%	1.2%
	Other	2.4%	1.2%
Stage	I	-	-
	II	-	2.5%
	III	60.0%	71.6%
	IV	40.0%	25.9%
Number of oncology treatment lines		3 (1)	3 (4)
Barthel		30 (40)	30 (45)
ECOG		3 (2)	3 (2)
MRC		3 (2)	3 (3)
SPPB		5 (8)	5 (10)

Regarding the socio-demographic results presented in Table 2, both groups had a median age of 67 years and a relatively balanced gender distribution (52.9% male, 47.1% female in the IG; 49.4% male, 50.6% female in the CG). The most prevalent oncological diagnosis was lung cancer, and the most common tumour stages were III and IV. The sample had a median of three treatment lines used.

### Functionality (IB)

The baseline results for this variable are presented in Table 2. Additionally, the scores by intervention group, as well as the statistical significance and effect size, can be found in Table 3. Statistically significant differences were observed between the IG and CG scores (*p* < 0.001; Table 3). The effect size (ES) for this parameter after the intervention was large (*d* = 0.887; 95% CI).

**Table 3:** Pre-intervention, post-intervention, and change scores for the variables under study (median and range for continuous data)

Variables	Intervention group (IG) (*n* = 87)		Control group (CG) (*n* = 83)		*p* value	Cohen’s *d*
	Basal assessment (T0)	Final assessment (T1)	Basal assessment (T0)	Final assessment (T1)		
Barthel	30 (45)	45 (60)	30 (45)	40 (65)	< .001	0.887
MRC	3 (3)	3 (3)	3 (3)	3 (2)	0.134	N/A
ECOG	3 (2)	3 (2)	3 (2)	2 (2)	< .001	0.561
SPPB	5 (10)	6 (9)	5 (8)	6 (8)	0.004	0.358

### Dyspnoea (MRC)

Table 2 shows the baseline results for this variable. Table 3 displays the scores by intervention group, along with the statistical significance and effect size. No statistically significant difference was found between the MI and CG scores (*p* = 0.134, *p* > 0.05) (Table 3).

### Physical performance (SPPB)

Table 2 shows the baseline results for this variable. Table 3 displays the scores by intervention group, along with the statistical significance and effect size. Statistically significant differences were found between the MI and CG scores (*p* = 0.004, *p* < 0.05) (Table 3). The effect size (ES) for this parameter after the intervention was medium (*d* = 0.358; 95% CI).

### Functional capacity (ECOG)

The baseline results for the functional capacity (ECOG) variable can be found in Table 2. Additionally, scores per intervention group, as well as statistical significance and effect size, can be found in Table 3. Statistically significant differences were observed between the MI and CG scores (*p* < 0.001; Table 3). The effect size (ES) for this parameter after the intervention was medium (*d* = 0.561; 95% CI).

Finally, regarding the study of correlations, we consider it crucial to perform a more precise analysis of both the proposed interventions and the associated symptomatology and clinical outcomes. Only studies with statistically significant positive results were deemed valid.

A positive correlation was observed between patient age and both MRC (*r* = 0.609) and ECOG (*r* = 0.599) scores. Conversely, a negative correlation was found with IB (*r* =  − 0.534) and SPPB (*r* =  − 0.627) scores.

In terms of functionality, a negative correlation was observed between IB and both ECOG (*r* =  − 0.862) and MRC (*r* =  − 0.853) scores, while a positive correlation was found between IB and SPPB scores (*r* = 0.812).

Regarding the level of dyspnoea, a negative correlation was observed between MRC and SPPB scores (*r* =  − 0.950).

For tumour stage, there was a positive correlation with the level of dyspnoea and MRC scores (*r* = 0.267). Additionally, there was a negative correlation with functionality, specifically IB scores (*r* =  − 0.373).

Lastly, concerning the number of drug treatment lines used, there was a positive correlation with the level of dyspnoea and MRC scores (*r* = 0.222). Furthermore, a negative correlation was found with functionality, specifically IB scores (*r* =  − 0.266).

## Discussion

The study found that a multimodal stress re-education programme, conducted by occupational therapists, nurses, and oncology physicians, improved functional (Barthel), physical performance (SPPB), and functional capacity (ECOG) in oncology patients with dyspnoea. However, not all individuals showed a direct relationship between these improvements and dyspnoea levels (MRC) compared to usual pharmacological treatment.

In recent years, there has been a growing interest in the symptomatic control of cancer patients due to the exponential increase in their survival rates [21]. This is because side effects, such as dyspnoea, become more prevalent as a result of the different treatments or the progression of the tumour process, as reported in the scientific literature [22].

It is essential that oncology patients experience a general improvement in their symptoms and are able to perform daily activities correctly during their hospital stay. This will have a positive impact on their autonomy once they are discharged from the hospital. This improvement not only benefits the patient but also their family, as it relieves them from assuming a caregiving role. In addition, this study proposes the prescription of individualised support products, such as walkers or standing frames, which have demonstrated greater benefits in terms of autonomy for cancer patients than recent scientific literature studies [23].

Upon closer analysis of the variables related to individuals’ levels of dependence (IB), it is evident that the implementation of the specific re-education programme for oncology patients has clear benefits. All individuals demonstrated statistically significant improvements after the programme’s implementation, with a large effect size. This is supported by similar studies, such as those conducted by Kenis or Serraino et al. [2, 24, 25]. In a comprehensive case-analysis study of 763 patients, it was observed that functional status (assessed with instrumental activities of daily living) was one of the main prognostic factors for cancer patient survival. Patients with better functional status had higher survival rates.

Regarding the levels of dyspnoea (MRC), there were no significant differences between the two groups studied. This could be attributed to the fact that the pharmacological treatment used in both groups effectively manages the symptom. However, this symptomatic relief does not extend to an improvement in the performance of daily activities, i.e. functionality. It is possible that individuals who have not received the effort re-education programme may experience fear of movement, which can lead to an acute exacerbation of symptoms. Additionally, patients may not be aware that they can resume their daily activities, which can also contribute to this fear. These factors can result in an increase in caregiver overload and a higher level of dependency, as noted in studies by Fernandez et al. [22].

Continuing the analysis of the variables under study, we observed clinical improvements in the functional capacity (ECOG) and physical performance (SPPB) of the individuals after the proposed programme, with a medium effect size. Furthermore, we observed a direct relationship between these improvements and the levels of functionality. This, together with a decrease in the intensity of dyspnoea, leads us to believe that these parameters may be directly related. Our findings are consistent with similar studies conducted by Magnuson [5] and Petrick et al. [26], as reported in the scientific literature. The importance of functional capacity and physical performance in patients diagnosed with an oncological disease is highlighted, along with the factors that negatively affect their deterioration. The study analysed a sample of *N* = 1434 cancer patients and found that their functional capacity decreased during the first year after diagnosis compared to a similar group without cancer. This highlights the importance of interventions aimed at maintaining or improving functional capacity as a sign of better health prognosis.

Finally, it should be highlighted that there is a direct relationship between poorer levels of functionality and greater symptoms of dyspnoea in patients who have received a greater number of lines of treatment or who have a more advanced tumour stage. The available evidence suggests that both increased comorbidities resulting from multiple lines of treatment and advanced tumour stages are associated with decreased functionality and increased risk of dependence in individuals. This is supported by studies in the scientific literature, including those by Galvin and Pergolotti et al. [6, 15]. The evidence indicates the necessity of employing interdisciplinary rehabilitation strategies from the perspectives of physiotherapy and occupational therapy to manage symptoms resulting from cancer. It is important to maintain an interdisciplinary approach, which is directly related to our study’s methodology.

Furthermore, it has been observed that the variable of age negatively impacts the levels of functionality, functional capacity, and dyspnoea. This observation is made without considering other variables such as tumour stage. Therefore, age can be considered a direct variable that worsens the general health status of the individuals under study, as noted in studies by Mohile and colleagues [27]. The study investigates whether a history of cancer is linked to particular geriatric syndromes in elderly individuals. It analyses a national sample of 12,480 community-dwelling seniors, estimating differences between those with and without cancer. The study found that elderly cancer patients have a higher prevalence of geriatric syndromes compared to those without cancer. This highlights the importance of prioritising interventions for older individuals to prevent associated comorbidities and improve prognostic factors for oncological diseases.

Finally, it is important to note the main limitations of this study. Participants could not be fully blinded due to the nature of the intervention. However, a recent meta-epidemiological study suggested that blinding is less important than commonly believed [28]. Additionally, the study was estimated to take 2 years, but the COVID-19 pandemic outbreak extended the recruitment time of the patients to be studied. As a limitation of the study and a consideration for future research, it is important to define how the progression in the level of difficulty or intensity of the stress re-education exercises was managed. This could be achieved through a predefined progression protocol or by using perceived exertion or difficulty scales.

In future studies, it is essential to implement this clinical practice during post-hospital follow-up, specifically after patients are discharged from the hospital. This approach ensures that patients continue to receive the necessary support and guidance as they transition back to their daily lives, promoting long-term benefits and adherence to the re-education programme. Additionally, it is crucial to address secondary symptoms commonly associated with oncological diseases, such as tumour asthenia and pain. By managing these secondary symptoms, we can enhance the overall well-being and quality of life for cancer patients, allowing them to more fully participate in and benefit from the proposed effort re-education programme. This comprehensive approach will enable all cancer patients, regardless of their specific conditions or symptomatology, to experience the advantages of improved functionality and reduced dyspnoea, ultimately leading to better health outcomes and a higher quality of life. By extending the focus of future studies to include these considerations, we can ensure a more holistic and effective treatment strategy for cancer patients.

## Conclusion

The study’s results underscore the positive impact of the exercise re-education programme on improving the autonomy of oncology patients with dyspnoea. The programme, implemented by an interdisciplinary team of nurses, occupational therapists, and oncology specialists, effectively addressed the primary objectives of enhancing respiratory symptoms and functionality. Additionally, the study investigated secondary objectives, including improvements in physical performance and functional capacity, and examined how socio-demographic factors influenced the intervention’s effectiveness. These findings demonstrate the programme’s comprehensive benefits in both symptom management and overall patient well-being.

## Data Availability

Sequence data that support the findings of this study have been deposited in the in the Repositorio Documental Gredos of the University of Salamanca. https://gredos.usal.es/
